# Transitioning to sustainable healthy diets: A model-based and conceptual system thinking approach to optimized sustainable diet concepts in the United States

**DOI:** 10.3389/fnut.2022.874721

**Published:** 2022-07-29

**Authors:** Prince Agyemang, Ebenezer M. Kwofie, Jamie I. Baum

**Affiliations:** ^1^Department of Biological and Agricultural Engineering, University of Arkansas, Fayetteville, AR, United States; ^2^Bioresource Engineering Department, McGill University, Sainte-Anne-de-Bellevue, QC, Canada; ^3^Department of Food Science, University of Arkansas, Fayetteville, AR, United States; ^4^Center for Human Nutrition, University of Arkansas System Division of Agriculture, Fayetteville, AR, United States

**Keywords:** sustainable diet concepts, decision-making, system thinking, optimized diets, plant-based dietary patterns

## Abstract

Food production and consumption are essential in human existence, yet they are implicated in the high occurrences of preventable chronic diseases and environmental degradation. Although healthy food may not necessarily be sustainable and vice versa, there is an opportunity to make our food both healthy and sustainable. Attempts have been made to conceptualize how sustainable healthy food may be produced and consumed; however, available data suggest a rise in the prevalence of health-related and negative environmental consequences of our food supply. Thus, the transition from conceptual frameworks to implementing these concepts has not always been effective. This paper explores the relative environmental and health risks associated with highly consumed food groups and develops a methodological workflow for evaluating the sustainability of diet concepts in the context of different health, socio-economic and environmental indicators. In addition, we apply the multi-criteria decision-making techniques (an integrated Analytic Hierarchy Process- Technique for order preference by similarity to ideal solution (AHP-TOPSIS) model) to examine the health and environmental impact of selected sustainable healthy diet concepts implemented in the United States. The principal findings indicate that adopting plant-based diet patterns would benefit the environment and the population's health. However, the up-scale, broad adoption and implementation of these concepts are hindered by critical bottlenecks. Hence we propose potential modification strategies through a conceptual system thinking approach to deliver optimized sustainable diet concepts to aid in the realization of the anticipated benefits of adoption/implementation.

## Introduction

In the last 50 years, global diets have been increasingly viewed as not healthy or environmentally sustainable. Instead, they are perceived to contribute to environmental degradation, natural resource depletion (for example climate change, biodiversity loss, increased freshwater consumption), and poor health (1).

Today, in the United States, over 78 million people are estimated to be obese, with the presence of associated chronic diseases such as coronary heart disease, stroke, and type 2 diabetes (2). These diseases are responsible for seven out of ten deaths in the United States, killing more than 1.7 million Americans each year. In addition, about 18.2 million adults have coronary artery diseases (3), while nearly 37 million Americans have diabetes, with nearly 90–95% of this attributed to type 2 diabetes (4). The high prevalence of obesity-related chronic diseases has been inexplicably linked with consumers' food choices and diet patterns.

From an environmental sustainability perspective, at the global level, our present diet pattern is responsible for over 70% of global freshwater withdrawals, occupies nearly 40% of arable land on the earth, and contributes over 30% of anthropogenic greenhouse gas emissions (GHGE) (5). As a result, it is the single greatest cause of eutrophication in water bodies (~78% contribution), loss of biodiversity, and air pollution through increased atmospheric fine particulate matter (6).

Acknowledging the existence of these challenges has led to the development of diverse views and concepts regarding sustainable healthy diets as an approach to tackling the trilemma existing between diets, human health, and environmental degradation. The term sustainable healthy diets encompass two dimensions, namely environmental sustainability and healthiness of the diet. This concept simultaneously integrates the environmental cost of food production and consumption with nutrient requirements in a socio-cultural and economic context within safe planetary boundaries (7). In other words, a sustainable healthy diet aims to provide a diet that promotes optimal growth and development and supports the physical, mental, and social wellbeing of all people at different life stages for the present, without compromising the capacity of the future generation (8).

Achieving a sustainable healthy diet in conjunction with the Sustainable Development Goals number 3 (Good health and wellbeing) and 12 (Responsible consumption and production) has resulted in the proliferation of several concepts to guide local, regional, and government agencies. Broadly, in the United States, the U.S Department of Agriculture, in collaboration with other stakeholders and international organizations, has made and continues to make massive investments across varying visions of the future to achieve sustainable and healthy diets for all Americans. Prominent among several concepts proposed are climate-smart agriculture, precision farming, Diet Guidelines for Americans, the Mediterranean diet, and the Eat Lancet Commissions diet recommendation of the Planetary Health Diet framework. Additionally, other diet patterns, sustainable agricultural production schemes, and global food initiatives have been fostered with varying resource consumption while improving human health and minimizing environmental impact (9). These concepts were formulated on the premise that a simple shift in diet behavior or pattern can lead to potential reductions in environment-health impact.

The research to date has confirmed the ramifications of sustainable diet concepts on either the health of people or one or more environmental indicators. This is exemplified by the work undertaken by Reinhardt et al. (10), where the authors expanded on the sustainability outcomes of U.S. diet patterns with a specific focus on environmental indicators such as land use, water consumption, energy use, and fertilizer use. Likewise, Mekonnen and Fulton (11) analyzed the consumptive water reductions for vegetarian, vegan and Healthy U.S diet styles. A cohort study by Orlich et al. (12) investigated the association of vegetarian diet and mortality, concluding that it is associated with reductions in all causes of mortality. Other studies have also reported on the socio-economic aspects sustainable diet concepts. Springmann et al. (13), reported that the adoption of flexitarian diets concept with less amount of meat and dairy reduced cost by 14%, while pescatarian diets increased cost by 2% in high income countries such as the US, UK and Australia. In the same study, the authors associate vegan diet concept as the most affordable as it reduced food cast by up to one third, with the vegetarian diet close to second among other diet concepts. In this regard high energy dense foods which can lead to health problems for people tend to be cheaper than highly nutritious foods such as fish and vegetables (14). Collectively, these studies indicate a relationship between different diet concepts and reduction in either health impact of people or environmental impact. Despite the relative abundance of these sustainability concepts, herein lies a conundrum. The transition from conceptual frameworks to implementing these concepts has not always been effective.

Therefore, in the present work, we apply indicators covering human health, environmental sustainability, and socio-economic dimensions of sustainable food systems to evaluate the implementation pathways of sustainable healthy diet concepts implemented in the United States of America. The result of the evaluation is input into an integrated Analytical Hierarchy Process-Technique for order preference by similarity to ideal solution (AHP-TOPSIS) decision-making framework to determine which: (1) indicator/criteria is of high priority to consumers (2) diet pattern concept has the highest nutrient adequacy and maximizes the potential of the prevention of diet-related chronic disease such as cardiovascular disease, obesity, and diabetes, and (3) diet pattern concept has the minimum environmental footprint based on different descriptors. Although some regionally oriented healthy diet concepts have different socio-cultural connotations and may not be considered as typical American diets, we leverage on the premise of their successful adoption in different parts of the world and their capacity to address nutritional inadequacy and environmental sustainability issues. In addition, their improved health outcomes reported in many epidemiological, cohort, and life cycle assessment studies address environmental concerns serve as the premise to conduct the AHP-TOPSIS analysis. The entire structure of the paper is as follows: Section two describes a rigorous four-step methodology for selecting a sustainable diet pattern and presents a workflow for implementing the AHP-TOPSIS decision model. The third and fourth sections discuss the study's significant findings by providing a brief overview of the historical diet-health-environment trilemma, highlighting the relative environmental and health risk of taking additional servings per day of 15 highly consumed food groups. The remaining part of the paper identifies barriers to implementing top-ranked diet concepts and provides modification strategies to selected case studies on different diet concepts.

## Methods

### Methodological framework

**Figure 1:**
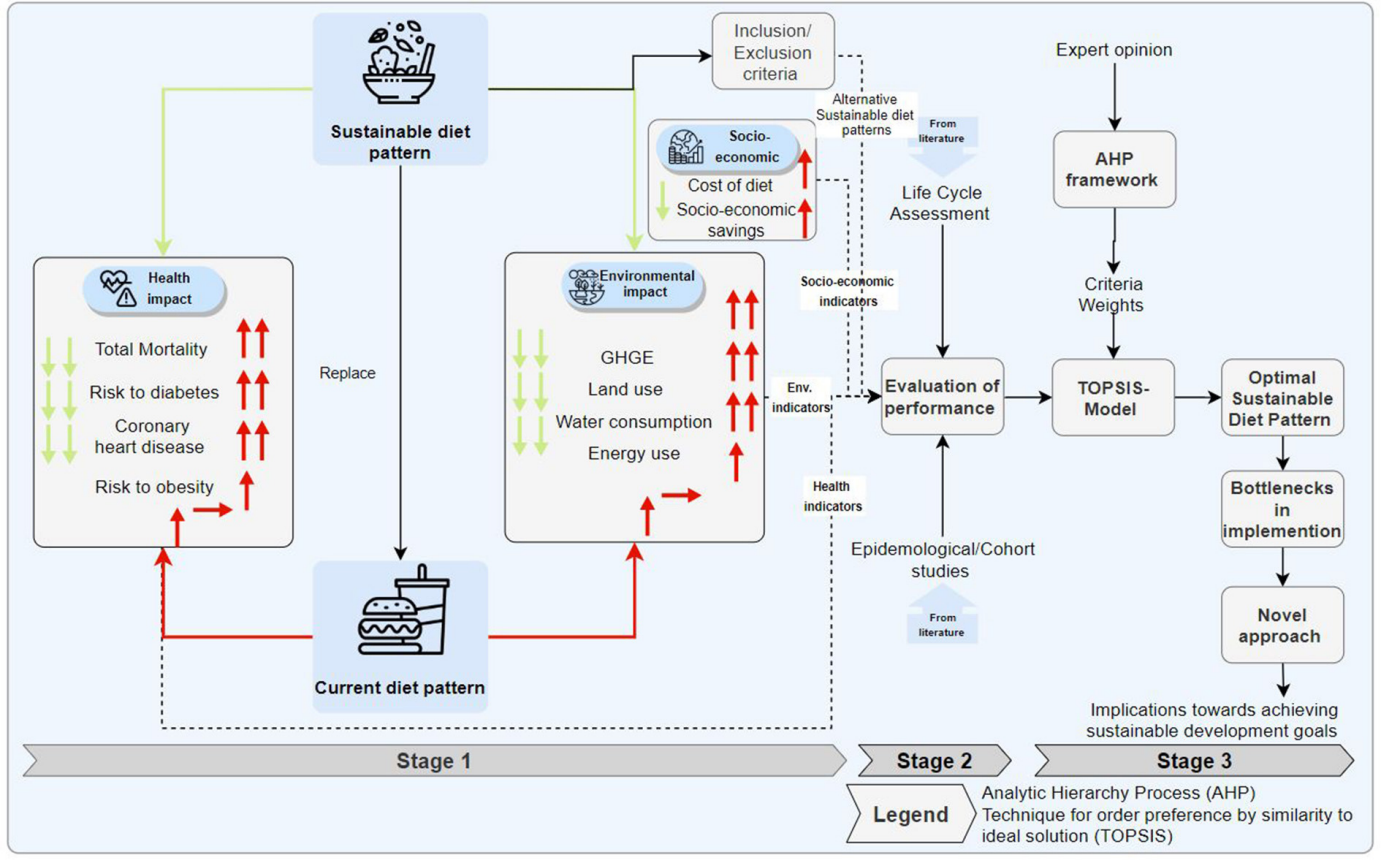
Conceptual framework for determining the performance of sustainable diet concepts in the United States.

It is evident that the sustainable (environmental) health diet trilemma that we are currently facing is due to the choice of the population under the influence of diverse factors such as increased income and urbanization. However, critical stakeholders have adopted and recommended many sustainable diet concepts due to the negative impact of people's choices on their health and the environment. Thus, to assess these diet concepts' efficiency and relative performance, this study adopts a methodological workflow to filter, evaluate, and seemingly predict the optimal effectiveness of implementing diverse sustainable and healthy diet concepts. Figure 1 presents the methodological framework adopted for determining optimal sustainable diets in the United States. In the first stage of the methodological framework, we highlight current diet choices' health and environmental impacts and present a historical trends of different impact categories such as GHGE, and overweight. Next, we identify several sustainable diet concepts and develop rigorous inclusion and exclusion criteria to determine which of them apply to the geographical region of focus. Next, we develop a metric to assess their current performance. The metric covers health, environment and socio-economic dimensions of sustainability. Finally, their performance results are input into an integrated AHP-TOPSIS framework to identify the concepts which could maximize the health, environmental gains and socio-economic gains. The AHP-TOPSIS relies on weights, which was computed taking into consideration expert opinions on the relative importance of the criteria used.

### Construction of an evaluation assessment index

According to Lancet Commission on Planetary Health, a shift in diet changes to a more sustainable diet can improve people's health and planetary health. For example, Lancet Commission analyzed healthy diets and determined that a shift toward such that's can prevent ~19–24% of total deaths that are diet related. Additionally, a shift to healthy diets was projected to decrease the prevented GHGE from the present baseline impact of 196% to 49–96% (7). Several other investigations into sustainable diet concepts have identified their correlation with improved health (including reducing mortality, diabetes, hypertension, heart diseases) in large American cohorts. Regarding the environmental impact, the literature shows that changing from a traditional Western diet to alternative diet patterns can reduce environmental footprint (15, 16). Therefore, to evaluate the impact of sustainable healthy diet concepts, we assembled a set of metrics that captures environmental sustainability, healthy diet benefits and economics. Table 1 presents a summary of the description of the metric and their respective objective in the context of sustainability.

**Table 1:** Metrics for evaluating sustainable diet concepts.

**Sustainability dimenstion**	**Sustainability metric**	**Description**	**Objective**	**References**
Health	Risk to diabetes	Measures the risk of diet concepts that affect the body's ability to produce insulin in cohort studies.	Minimized	(17)
	Prevention of coronary heart diseases	Measures the diet concept's risk in relation to coronary heart diseases in cohort/epidemiological studies.	Minimized	(18)
	Risk to mortality	Estimates the association of mortality to diet factors	Minimized	(19)
	Risk to obesity	Measures the association of different diet concept intake to the prevalence of obesity	Minimized	
	Total cancer	Measures the risk reduction to cancer from the consumption of different diet patterns	Minimized	(20)
Environment	GHGE reduction (kg CO_2_eq/capita/year)	It is an adjusted indicator that includes CO_2_, N_2_O, and CH_4_	Minimized	(21)
	Agricultural Land use reduction (m^2^/capita/year)	Measures the aggregated land use of the different types of agricultural production e.g., Pasture, cropping	Minimized	(22)
	Water consumption (L/capita/day)	Measures the amount of groundwater evapotranspired by crops or incorporated into the product during growth and processing.	Minimized	(11)
	Energy consumptions	Measures the amount of energy consumed during agricultural product of sustainable diets.	Minimized	(23)
Socioeconomic	Average cost of a healthy diet, €/day	Measures the cost of adherence to diet patterns per day	Minimized	(24)
	Socio-economic savings to society	Measures the savings through health and environmental improvements of consuming sustainable diets	Maximized	

### Criteria for inclusion of implementation case studies

#### Eligibility criteria

Before evaluating the sustainable diet concepts, we developed rigorous four-step inclusion and exclusion criteria for observational and epidemiological trials that have studied the association between diet patterns on either health or the environment. In the first step, we searched the literature to identify diet patterns and studies on health, and environmental assessment, which focused on U.S. only. Next, we set a minimum threshold of five studies that have reported implementing the initially sampled diet pattern. This step ensured substantial historical evidence of its effectiveness against real environmental and health pressures. Later, a cut-off criterion that considered the sample size, population demography, and duration of the implementation case studies was applied. In each sub-inclusion/exclusion criteria, a threshold of 5,000 participants, including women and children was set. A 4-year study period and monitoring were used to eliminate studies that did not meet the above requirement. The overall cut-off contribution for each sub-criterion was set at 60%. Aside from this, we checked the availability of data on environmental impact assessment and health risk results associated with each diet.

#### Data grid

The data used in analyzing the health and environmental burden of American diet shifts and lifestyles was obtained from FAOSTAT, “Our World in Data”, and “World bank” (25, 26). In addition, we constructed a new database using the available literature on health impact and environmental impact assessment of different diet patterns. Data on the literature used, including the publication year, study country, primary health, environmental and socio-economic outcomes, are shown in the Supplementary material. Where no data was available during the assessment, proxies from high-income countries such as Canada, Sweden, and the United Kingdom were adopted. Additional data for assessing and analyzing the effectiveness of the different diet concepts were from the work of Clark, Springmann (27).

### Multi-criteria decision-making method

According to Johnston et al. (28), a sustainable diet promotes environmental and economic stability through low impact and affordable foods while simultaneously improving the population's health by providing adequate nutrition and reducing the risk of diseases. A systematic methodological evaluation of current sustainable healthy diet patterns and their effectiveness in addressing regional and global objectives in the health and environmental context is required to realize these objectives. This is partly because of the complex interactions between health, environment, and socio-economic drivers such as consumer demands. Therefore, sustainable decision-making should integrate Multi-criteria Decision-Making (MCDM) tools to ensure healthy diets delivered from a sustainable food system is achieved for nearly 10 billion people by 2050. Table 2 summarizes MCDM and their respective methods.

**Table 2:** Categories for classifying multi-criteria decision making methodologies [extracted from (29)].

**Categories**	**Methodology**
Multi-attribute utility and value theory	Analytic hierarchy process (AHP)/analytical network process (ANP); fuzzy set methodology; gray relational method
The multi-objective mathematical programming	Constrain programming; linear programming; goal programming
Non-classical method	Fuzzy set methodology
Elementary aggregation method	Weighted sum method; weighted product method
Complex aggregation method	Analyse and synthesis parameters under information deficiency (ASPID)
Distance-to-target approach	Technique for order preference by similarity to ideal solution (TOPSIS); gray relational analysis; data enveloping analysis
Direct ranking (high dependence on decision-maker)	Stepwise expert judgment; Delphi; scoring method
Outranking method	Elimination and choice translating reality (ELECTRE I, I.S., II, III,); preference ranking organization method for enrichment evaluation (PROMETHEE I, II)

#### Overview of MCDM method employed in this study

MCDM methods have been widely applied to different sectors, including social, economic, industrial, biological systems, and renewable energy systems (30). Contrary to the single criteria decision-making approach, MCDM employs a multi-attribute/criteria approach to obtain an integrated result for the decision-maker. It is important to mention that not all MCDM methods are the same; while some incorporate certain features, others disregard and are limited in different perspectives. More often than not, the choice of technique is dependent on the availability of data, knowledge of the method, the context of the problem, and the software to implement the method. One of the most well-known, highly adopted, and simplest subjective and objective MCDM methods in food system evaluation include Analytical Hierarchy Process (AHP) and technique for order preference by similarity to ideal solution (TOPSIS). AHP provides a straightforward and flexible model to address problems. When there are multiple conflicting criteria, it becomes expedient to adopt such a method to achieve a consensus.

#### Relative weight determination using analytical hierarchy process

In general, MCDM requires an evaluation of ***m*** criteria against ***n*** alternatives, as presented in Equation (1).


(1) $$\begin{array}{l} \;criteria\;C_{1}\;C_{2}\;\ldots \ldots .C_{m}\\ \left(weights\;w_{1}\;w_{2}\ldots \ldots .\;\ldots w_{n}\right)\\ X=\;\begin{array}{c} A_{1}\\ \vdots \\ A_{n} \end{array}\;{\left(\begin{array}{ccc} x_{11} & \cdots & x_{1n}\\ \vdots & \ddots & \vdots \\ x_{m1} & \cdots & x_{mn} \end{array}\right)}_{m\times n} \end{array}$$

Where *A*_*i*_ are the alternative sustainable diet concepts, *x*_*ij*_ is the performance of *j-th* criteria of the *i-th* alternative sustainable diet concept and *w*_*j*_ is the weight of criteria. This weight is obtained by employing the A.H.P. method. Saaty and Ramanujam first introduced the A.H.P. method in a seminar work to provide a comprehensive evaluation model of different criteria relevance in problems. The technique uses a pair-wise comparison model that first defines the objective of the decision problem, decomposes to other criteria and sub-criteria, depending on its complexity, and determines unique weights for each criterion (30, 31).

#### Ranking of sustainable diet concepts using technique for order preference by similarity to ideal solution

With an *A*_*i*_ number of alternatives, the ranking to obtain the optimal sustainable diet concept is achieved using the TOPSIS model. TOPSIS is a practical and valuable method for ranking and selecting several possible alternatives through measuring Euclidean distances. It is based on the concept that the chosen alternative should have the shortest distance from the positive ideal solution (P.I.S.) in a geometric sense.

#### Premises for the AHP-TOPSIS modeling of sustainable diet concepts

As mentioned earlier, not all MCDM methods incorporate certain features. The AHP method cannot capture uncertainties and determine alternative ratings in decision-making. This weakness is complemented by TOPSIS, making the use of an integrated AHP-TOPSIS technique a more robust approach to decision making.

Assuming we have ***m*** number of criteria, the AHP model can be implemented as follows:

***Step 1:*** This involves developing a hierarchy structure that describes the goal, alternatives, criteria, and sub-criteria for the comprehensive evaluation.

***Step 2:*** Here we construct a pair-wise comparison for the criteria and alternatives concerning the decision-making objective. Table 3 shows the relative pair-wise comparison connotations that express each criterion's importance based on the decision makers' discretion.

**Table 3:** Definition of the intensity of qualitative and quantitative score for criteria weight determination.

**Intensity of weight**	**Definition**	**Explanation**
1	Equal importance	This implies that two criteria have equal importance to the objectives.
3	Moderate importance of one over another	The judgment slightly favor one over the other
5	Strong importance	The judgment strongly favor one over the other
7	Extreme importance	The judgment is very strongly favored one over the other
9	Absolute importance	The judgment is of absolute importance over the other

It is important to mention an instance where intensity adjusted weights such as 2, 4, 6, and 8 can express intermediate importance between criteria. The matrix was constructed in accordance with the recommendations of (29) to extract a decision matrix.

***Step 3:*** The second step is repeated for each criterion, and then the priority of alternatives is acquired by accumulating the weights. Next, a statistical technique, arithmetic mean method is adopted to construct a vector *W* = [*W*_1_, *W*_2_, …*W*_*N*_] that represents the weight of each criterion in a pair-wise comparison matrix M presented in Equation (1). Each element in column *j* of matrix *M* is divided by the sum of entries in the j column. This step generates a new matrix called the Normalized matrix (*A*_*norm*_). It is important to highlight other statistical techniques such as the characteristic root method, and the least square method can be employed to estimate the weights.


(2) $$\begin{array}{r} \text{M}=\;\left[\begin{array}{ccc} \frac{{\text{C}}_{1}}{{\text{C}}_{1}} & \cdots & \frac{{\text{C}}_{1}}{{\text{C}}_{\text{N}}}\\ \vdots & \ddots & \vdots \\ \frac{{\text{C}}_{\text{N}}}{{\text{C}}_{1}} & \cdots & \frac{{\text{C}}_{\text{N}}}{{\text{C}}_{\text{N}}} \end{array}\right] \end{array}$$

***Step 4***: The comparison matrix (Equation 1) obtained in step 3 is subjected to a consistency check to validate the results' soundness. A consistency ratio of 10% or 0.1 was set. This involves determining the maximum eigenvalues and consistency index by using Equations (2) and (3), respectively. One advantage of the consistency ratio is that it eliminates the problem of disagreements in individual judgments.


(3) $$\begin{array}{r} \lambda_{max}=1/n\overset{n}{\underset{i=1}{\sum }}\frac{i^{th}entry\;in\;AW^{T}}{i^{th}\;entry\;in\;W^{T}} \end{array}$$

*Where:* λ_*max*_ = *maximum Eigen value*

*n* = *number of attributes*

*A* = *pair-wise comparison matrix*

*W* = *The estimate of the decision-makers weight*

Nevertheless, the consistency is checked by comparing the Consistency Index (CI) to the Random Index (R.I.) for the appropriate value of n, used in decision-making (30). If (CI/RI) < 0.10, the degree of consistency is satisfactory, but if (CI/RI) > 0.10, serious inconsistencies may exist, and the results produced by AHP may not be meaningful.


(4) $$\begin{array}{r} CI=\frac{\lambda_{max}-n}{n-1} \end{array}$$

**Figure 2:**
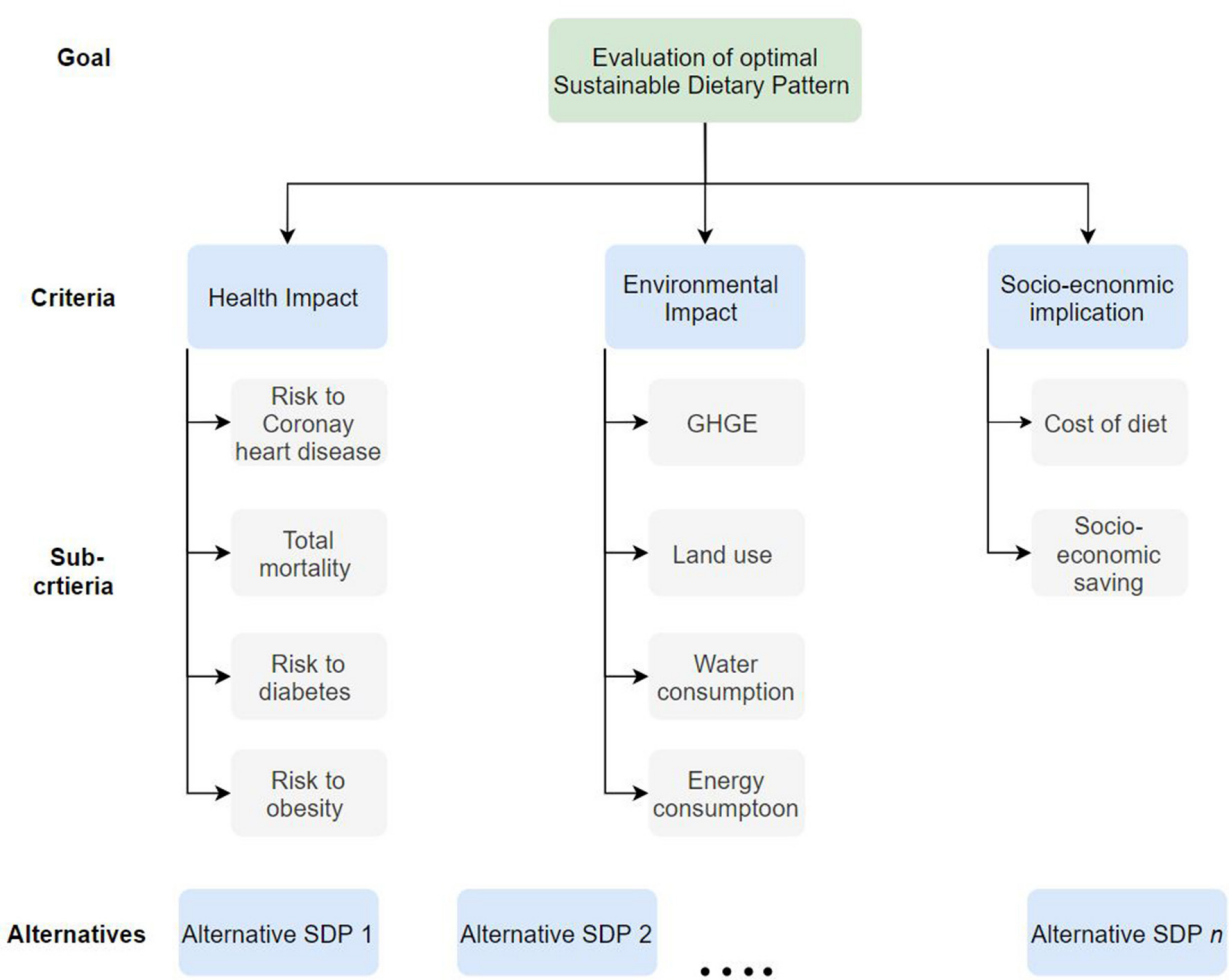
Hierarchical breakdown for assessing the performance of diet concepts.

Figure 2 presents the hierarchical decomposition of the decision-making problem. It summarizes the overall objective: to determine an optimal sustainable diet pattern, the criteria, and sub-criteria used to evaluate the sustainable diet concepts.

Assuming we have ***n*** number of alternative sustainable diet concepts, the TOPSIS ranking for the alternatives can be achieved through the following:

***Step 5:*** Construct the normalized decision matrix

In this step, the different attributes' dimensions are transformed into a non-dimensional attribute to allow comparison across the attributes. Using the method represented in Equation (3), the matrix (_*x*_*ij*_)*m* × *n*_ is normalized to *R* = (_*r*_*ij*_)*m* × *n*_ which takes the form shown below


(5) $$r_{ij}=\frac{x_{ij}}{\sqrt{\sum_{j=1}^{m}x_{ij}^{2}}}\;i=1,2,\;\ldots .,\;m;j=1,2,\;\ldots ,\;n$$

(6) $$R=\left(\begin{array}{ccc} r_{11} & \cdots & r_{1n}\\ \vdots & \ddots & \vdots \\ r_{m1} & \cdots & r_{mn} \end{array}\right)$$

***Step 6:*** Construct the weighted normalized decision matrix

With the normalized decision matrix (R) computed from the previous step, the weighted matrix W from the A.H.P. method is integrated into the R. This results in a matrix calculated by multiplying each column of R with its associated weighted matrix W *represented in* Equation (4).


(7) $$\begin{array}{r} V_{ij}=w_{j}\times r_{ij}\;where\;i=1,\;2,\ldots .,n \end{array}$$

This computation results in a new matrix V, which is represented below


$$\begin{array}{l} V=\left[\begin{array}{ccc} v_{11} & \cdots & v_{1n}\\ \vdots & \ddots & \vdots \\ v_{m1} & \cdots & v_{mn} \end{array}\right]=\left[\begin{array}{ccc} {w_{1}r}_{11} & \cdots & w_{n}r_{1n}\\ \vdots & \ddots & \vdots \\ {w_{1}r}_{m1} & \cdots & {w_{n}r}_{mn} \end{array}\right] \end{array}$$

***Step 7:*** Determine the ideal and negative ideal solutions

In this process, two artificial alternatives *A*^+^ (the ideal positive alternative) and *A*^−^ (the ideal negative alternative) are defined as:


$$\begin{array}{l} A^{+}=\left\{v_{1}^{+},v_{2}^{+},\ldots ..,v_{n}^{+}\right\}=\left\{\left(\underset{j}{\max}v_{ij}|i\in I^{{}^{\prime }}\right),\left(\underset{j}{\min}v_{ij}|i\in I^{{}^{\prime \prime }}\right)\right\}\\ \;i=1,2,\ldots ..,m;j=1,2,\ldots ,n.\\ A^{-}=\left\{v_{1}^{-},v_{2}^{-},\ldots ..,v_{n}^{-}\right\}=\left\{\left(\underset{j}{\min}v_{ij}|i\in I^{{}^{\prime }}\right),\left(\underset{j}{\max}v_{ij}|i\in I^{{}^{\prime \prime }}\right)\right\}\\ \;i=1,2,\ldots ..,m;j=1,2,\ldots ,n. \end{array}$$

Where *I*′ is related to benefit attributes, and *I*″ is related to cost attributes

***Step 8:*** Achieve the remoteness of all choices from *A*^+^ and *A*^−^

In the process, the separation measurement is done by calculating the distance between each alternative in *V* and the ideal vector *A*^+^ using the Euclidean distance, which is given as Equations (5) and (6)


(8) $$\begin{array}{ccc} D_{i}^{+} & = & \sqrt{\sum_{j=1}^{n}{\left(v_{ij}-v_{j}^{+}\right)}^{2}i=1,2,\ldots ..,m} \end{array}$$

(9) $$\begin{array}{ccc} D_{i}^{-} & = & \sqrt{\sum_{j=1}^{n}{\left(v_{ij}-v_{j}^{-}\right)}^{2}i=1,2,\ldots .,m} \end{array}$$

Where $D_{i}^{+}$ and $D_{i}^{-}$ are the Euclidean distance from the ideal best and ideal worst, respectively. At the end of this, two quantities, namely $D_{i}^{-}$ and $D_{i}^{+}$ for each alternative has been counted, representing the distance between each alternative and both (the ideal and the negative ideal).

***Step 9***: Determine the relative closeness to the ideal solution using Equation (9).


(10) $$\begin{array}{l} \begin{array}{ccc} CC_{i}^{{}^{*}} & = & \frac{D_{i}^{-}}{D_{i}^{-}+D_{i}^{+}} \end{array}\\ \;\begin{array}{ccc} i & = & 1,2,\ldots .,\;m \end{array} \end{array}$$

Where $CC_{i}^{*}$ Is the performance score.

**Figure 3:**
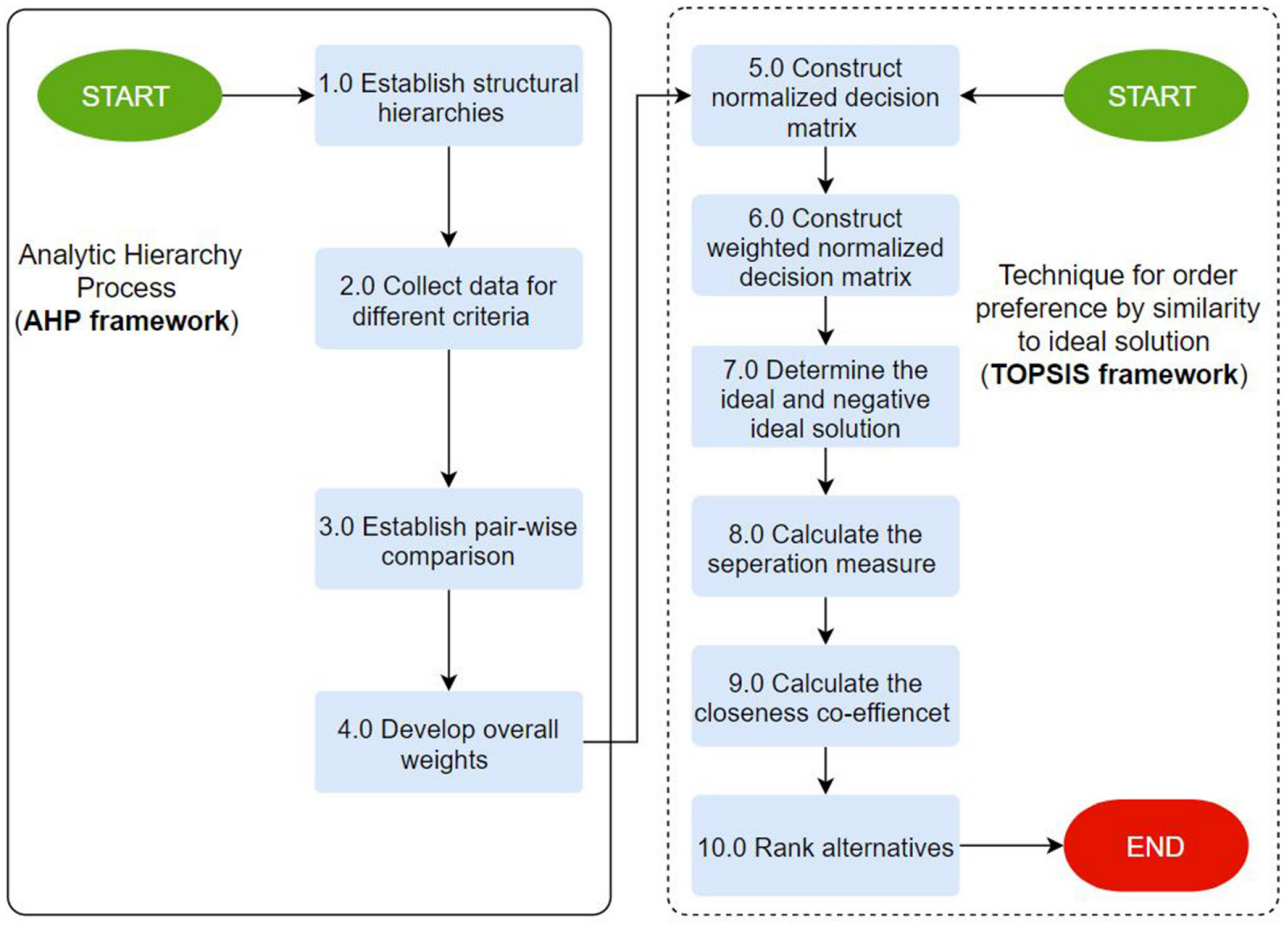
An integrated AHP-TOPSIS from selecting optimal diet concepts in the United States.

***Step 10:*** Rank the alternatives according to relative closeness to the ideal solution. All alternatives (sustainable diet patterns) are based on the performance score in this step. Figure 3 presents the continuous workflow of the integrated AHP-TOPSIS framework that is adopted to evaluate sustainable diet concepts. Step 1 to 9 presented above provide an elaboration of the components of the workflow.

## Results

### Historical interactions between the diet, health, and environmental trilemma

**Figure 4:**
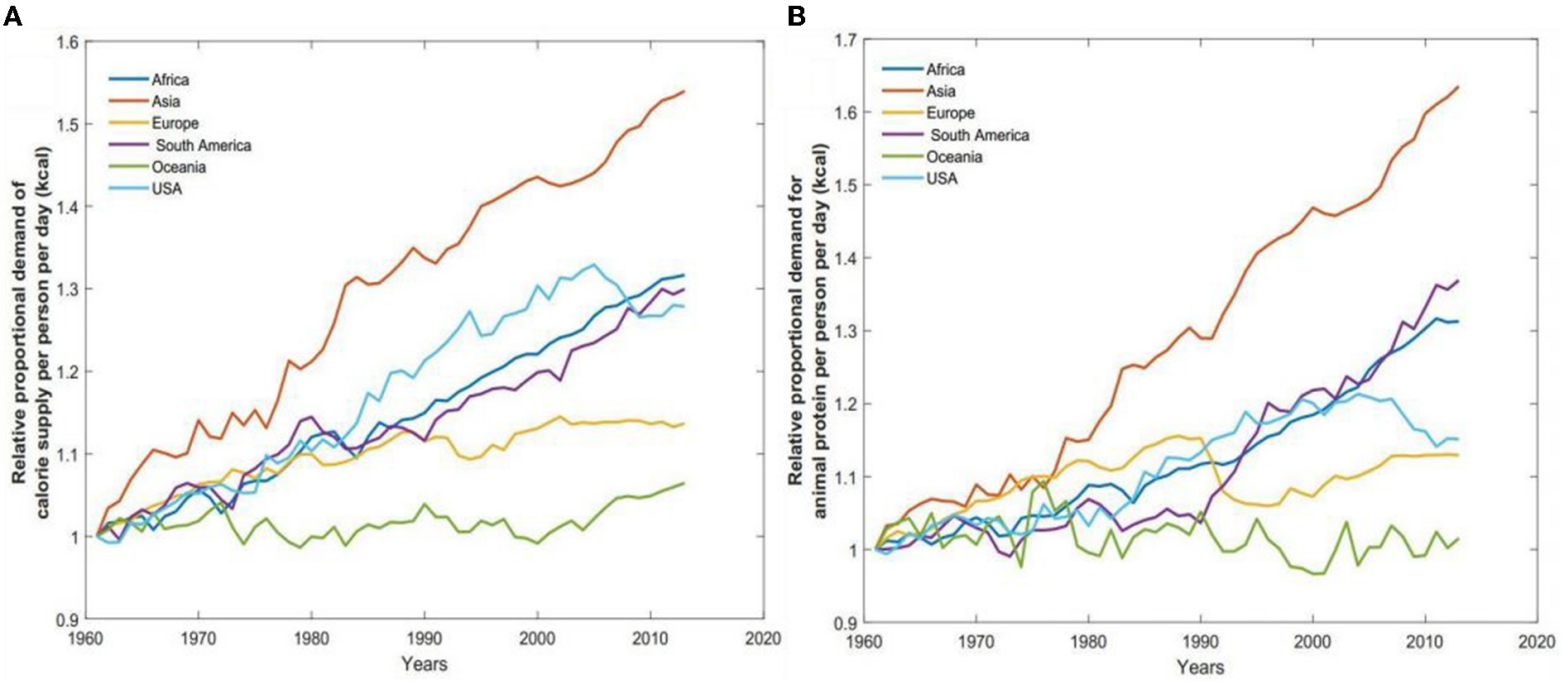
Relative proportional demand in animal-based protein and calorie be capital per day. **(A)** Estimated daily calorie supply per capita across all regions from 1961 to 2013. **(B)** Daily animal protein demand in each region from 1961 to 2013.

This section explores how United States diet shifts and lifestyles have rapidly worsened the health-environmental burden in the country. Figure 4 presents historical trends of the relationship between food demands in different geographic regions against United States. From Figures 4A,B, we observe that over the last 60 years, there has been a relative proportional increase in per capita calorie supply and a consistent increase in demand for animal-based protein across the globe. For the case of calorie demand per capita, the increase has been most significant in United States, Asia, South America, and Africa. We observe that the per-capita rate of increase has been 19.56 and 17.94% for United States and Asia, respectively. For instance, the calorie demand has increased by 53.96, 31.66, 30, and 27.84 % in Asia, Africa, South America, and United States. However, the relative rate of increase on an annual basis within that same time frame is 17.94, 12.35, 1.66, and 19.56%, respectively. This indicates that despite the significant increase in demand in Asia, Africa, and South America, the annual increase in consumption in America is more significant. Interestingly, in the 21st century, while other regions continue to experience an increase in demand for calories, United States has experienced a sudden plateau.

Similarly, we observe that the demand for protein (animal-based foods) has followed a similar pattern. Demand in Asia, South America, Africa, and United States is ~63.5, 36.9, 31.27, and 28% respectively. However, we observed 58.06, 42.79, and 40.57% annual increases in protein demand in Asia, South America, and United States, respectively. It is interesting to note that the demands in Europe have consistently decreased between 1990 and 2013. Likewise, United States has consistently experienced an increase in demand since 1960, but in the last decade has suddenly plateaued.

**Figure 5:**
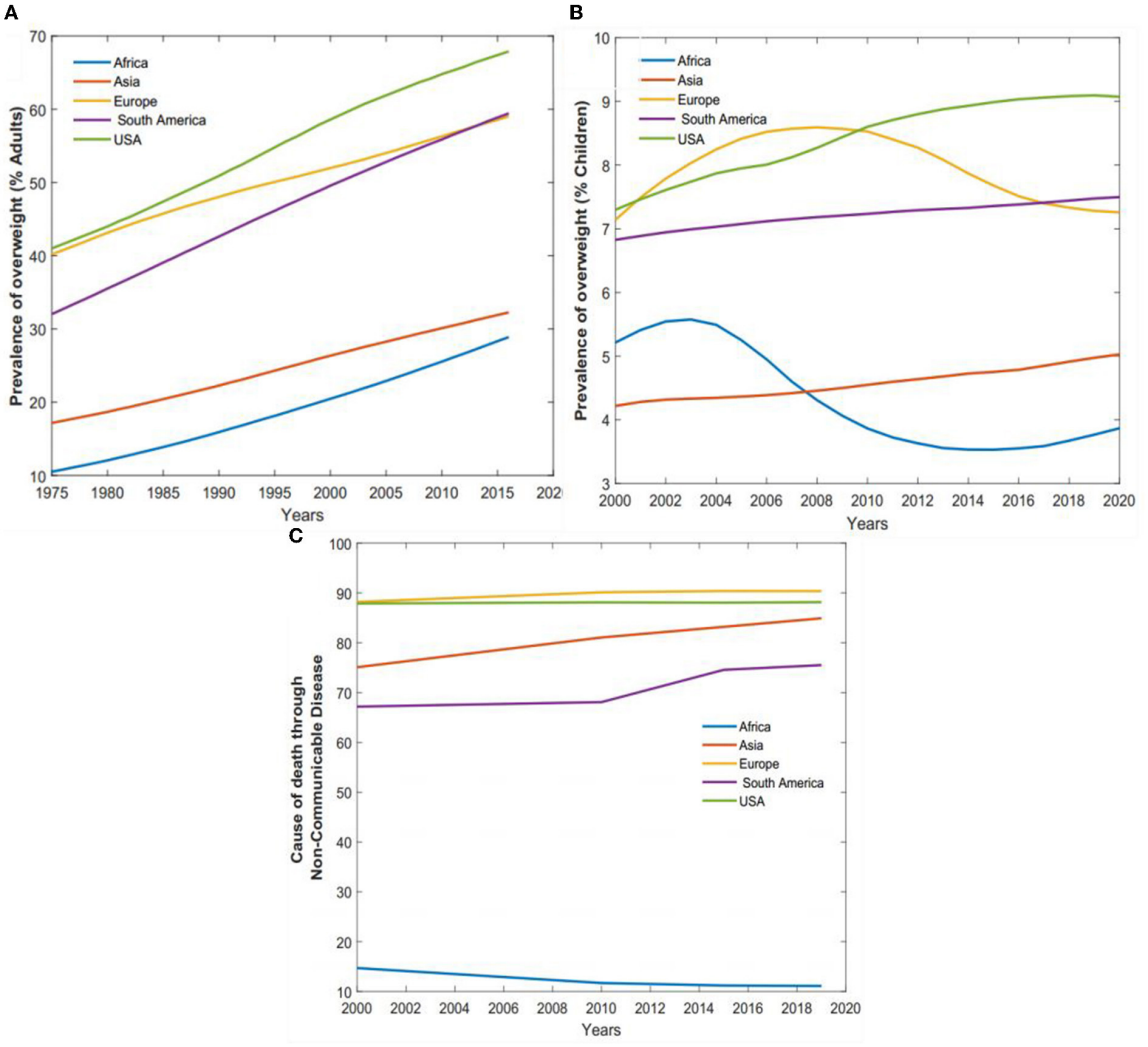
The prevalence of health-related implications of a consistent intake of unhealthy food over time. **(A)** Percentage prevalence of overweight in adults from 1975 to 2016. **(B)** Percentage prevalence of overweight in children under 5 years from 2000 to 2020. **(C)** Percentage total cost of death due to non-communicable diseases from 2000 to 2019.

Previous studies by many researchers such as (32–34) have established a relationship between diet-related diseases such as diabetes and heart diseases and obesity and the consistent shift in diet toward an increased reliance on high calorie, animal-based, highly processed foods and sugar-sweetened beverages. As a result, the prevalence of overweight in adults has increased worldwide. Figure 5 illustrates regional increase in overweight among adults and children and the percentage of total death associated with non-communicable diseases. In the United States, overweight predominance has risen from 41.00 to 67.90% between 1975 and 2015, representing the single most significant increase globally. On the contrary, Asia has experienced a relatively lower increase from 17.00 to 32.26%. Also, in developing regions such as Africa, we observe a moderate rise from 10.51 to 28.89%. In Europe, we observe 39.20 to 58.64%. The increase in the prevalence of overweight consequently influences the global burden of disease and death associated with diet, which has significantly increased from 60.80% in 2000 to 73.63% in 2019. Within this same timeframe, the United States has been responsible for nearly 17.80–14.51% of such incidence.

**Figure 6:**
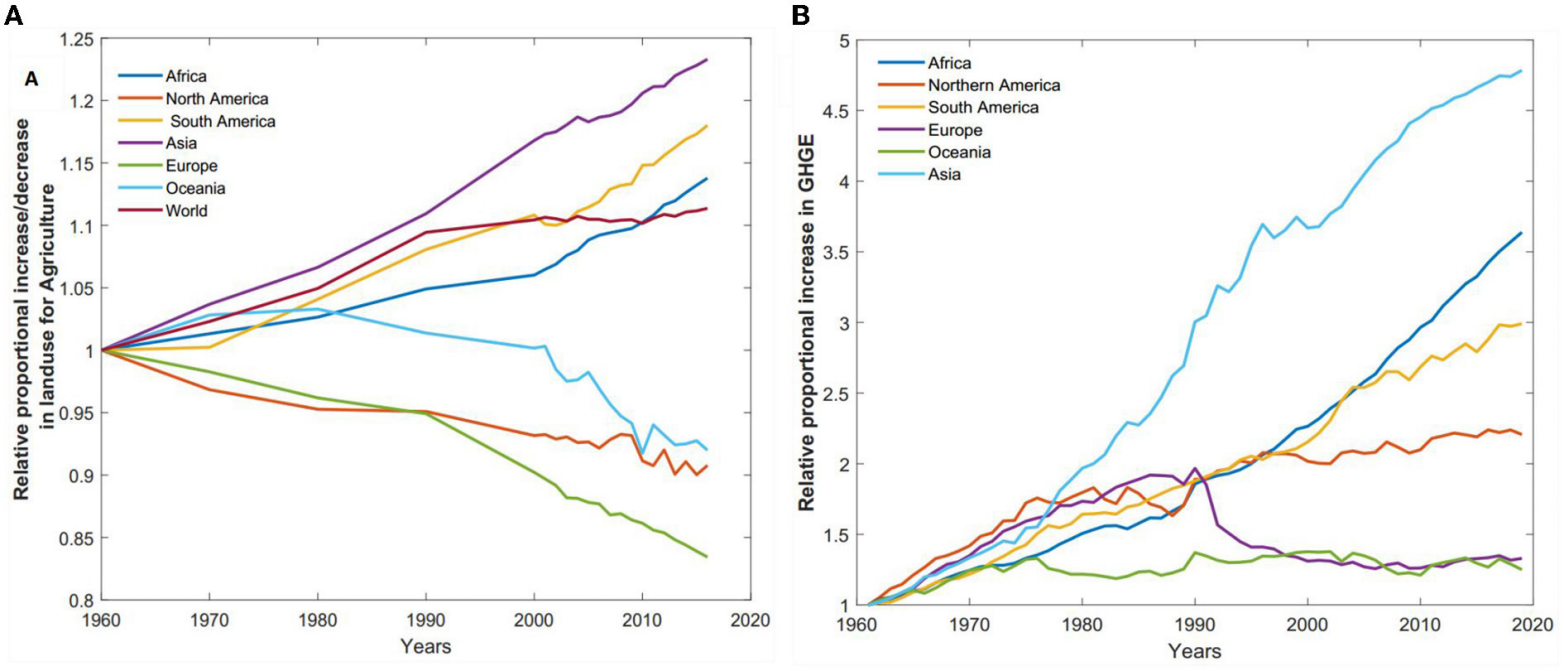
Relative proportional increase/decrease of agricultural land use and GHGE. **(A)** Relative proportional increase/decrease of land use from 1960 to 2016. **(B)** Relative proportional increase in GHGE from 1961 to 2019.

Aside from the health impact, the consequences of diet choices extend to impact our environment (Figure 6). This is evidenced in Figures 6A,B where we observe a relative proportion of agricultural land use and its corresponding GHGE. Globally, our arable land use for crop production has increased by more than 11.36%. Interestingly, we observe a continuous decrease in land use in Europe, and Oceania, probably due to technology 4.0 into agricultural production, which results in a steady reduction in GHGE from these regions. Notwithstanding, we see a consistent rise in the United State. In general, greenhouse gas emissions from agricultural production have increased since 1961. It has been the largest in developing countries such as Asia (378% increase since 1961) and Africa (263% increase since 1961). The United States has experienced nearly about 120% increase within that same timeframe.

### Relevant environmental and health risk of highly consumed food group

Customarily, we decide every day on what to eat, considering the taste, nutritional benefits, safety, and, perhaps more recently, the environmental friendliness of the food. Recent evidence suggests that nearly 40% of the world's annual mortality is attributed to diet-related diseases such as stroke, coronary heart disease, type 2 diabetes, total cancer, and many others (35, 36). Lozano et al. (37) identified that nine out of the top fifteen risk factors for the consistent annual increase in global mortality were associated with diet choices. This implies that the choice of food and its corresponding quantity we take in is a significant determinant of our health and the sustainability of our environment. Our diets today pose a high risk to ill-health and threaten the achievability of sustainable development goals.

**Figure 7:**
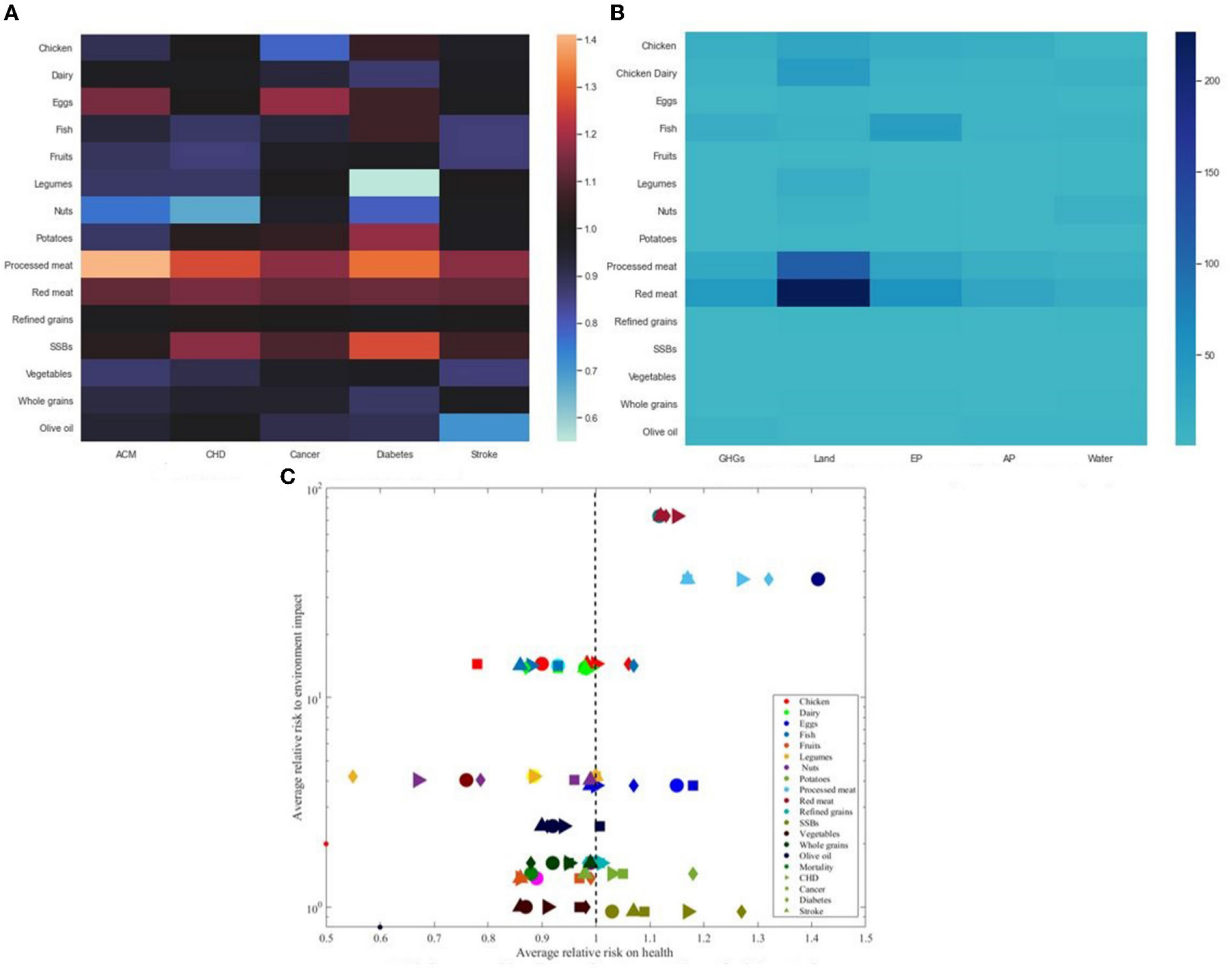
Relative Risk to diseases and environmental impact of 15 highly consumed food groups. **(A)** Relative risk to disease for different food groups. **(B)** Relative risk to environmental damage for different food groups. **(C)** Relative average risk to disease and environmental impact for different food foods..

This section explores the relative environmental and health risks associated with 15 highly consumed food groups. The health outcomes considered include type 2 diabetes, stroke, coronary heart disease, total cancer, and mortality. In contrast, the environmental concerns include GHGE, land use, eutrophication potential, acidification potential, and water consumption. Figure 7 presents the relative risk to diseases and the environmental impact of 15 food groups. The data used was obtained from (37).

It is essential to mention that if the relative risk is >1, then the consumption of an additional serving of such food group poses an increased risk to disease. Likewise, relative risk <1 is correlated to a lower or decreased disease risk. From Figure 7, we observe that the additional consumption of vegetables, wholes, fruits, legumes, and fish are associated with reducing diseases risk to coronary heart disease, diabetes, total cancer, stroke, and total mortality. In addition, there are correlated to a lower environmental impact. Conversely, red meat and unprocessed meat consumption are associated with increased risk and environmental impact. This implies shifting diet behaviors toward food groups with lower environmental and health risks has the potency to reduce the diet-health and environmental implications. The following section describes the methodology developed to select and evaluate sustainable diet concepts.

### Selected sustainable diet concepts

Table 4 presents the results after applying the inclusion-exclusion criteria described in section results. Diet patterns with (−) signs imply limited implementation cases and available data to support the claims of lower environmental footprint and health-related issues in the United States. Although there is literature to confirm these diet recommendations as sustainable diet concepts, there was little evidence from clinical or epidemiological studies and life cycle assessment or environmental impact modeling or input-output analysis on the impact of adopting these sustainable diets. In addition, the majority of results the authors found were focused on European countries, which is outside the scope of this study. Also, the authors discovered that many of the sustainable diet concept analyses were conducted in high-income countries with well-established diet guidelines.

**Table 4:** Selected sustainable diet concepts after the four-step inclusion/exclusion criteria.

**S/N**	**Sustainable diet concept**	**Step 1**	**Step 2**	**Step 3**	**Step 4**	**Status**
1	The mediterranean diet pattern	√	√	√	√	*
2	Healthy planetary diet (EAT-Lancet pattern)	×				–
3	Healthy vegetarian diet pattern	√	√	√	√	*
4	Atlantic diet pattern	√	√	√	×	–
5	Diet approach to stop hypertension (DASH diet)	√	√	√	√	*
6	Pesco-vegetarian	√	√	√	√	*
7	The healthy nordic diet pattern	√	√	√	×	–
8	Paleolithic diet	√	√	√	×	–
9	Tradition persian medicine diet	√	×			–
10	Vegan diet	√	√	√	√	*
11	The healthy U.S style diet pattern	√	√	√	√	*
12	Chinese diet pattern	√	√	√	×	–
13	Western diet concept	√	√	√	×	–
14	Spanish diet pattern	√	√	√	×	–
15	Provegetarian food pattern	√	√	√	√	*
16	Pescatarian diet	√	√	√	√	*
17	Flexiterian diet	√	√	√	√	*

(Where S/N refers to Serial Number, ^*^refers to a selected diet concept , – refers to rejected diet concept after comparing against the inclusion and exclusion criteria).

It is important to mention that other diet patterns have proportional magnitude variations in the quantity of animal and plant-based foods. Typical examples of diet patterns identified from the literature include meat partially replaced by plant-based food, meat partially replaced by mixed foods, meat + dairy partially replaced by plant-based foods (38, 39). However, because of the high degree of variations and the absence of extensive literature on such diet concepts, they were excluded from the variety of sustainable diet patterns used in this study.

### Overview of diet concepts

From Section Selected sustainable diet concepts, we observe that nine different sustainable diet patterns have been selected. Therefore, this section provides a high-level overview of the respective diet patterns and their corresponding food groups.

#### The mediterranean diet pattern

Global interest in this diet pattern began in the early 1960s when it was observed that seven countries near the Mediterranean Sea had a lower incidence of chronic disease. The diet is often associated with countries bordering the Mediterranean Sea, including Spain, France, Italy, Greece, Turkey, Northern Africa, Middle Eastern, and Balkan countries. This diet pattern has been described as (i) daily consumption of unrefined cereals and cereal products, vegetables, fruit, olive oil, dairy products, and red or white wine; (ii) weekly consumption of potatoes, fish, olives, pulses, and nuts and eggs and sweets and monthly consumption of red meat (40).

#### Healthy vegetarian diet pattern

This diet pattern is devoid of any food product that contains meat or fish. In addition, diets containing poultry, seafood, and f lesh of any animal are strictly prohibited.

#### Diet approach to stop hypertension (DASH diet)

This diet concept was introduced to control the risk of hypertension. The essential ingredients peculiar to this diet includes fruit, vegetables, and low-fat dairy products, including whole grains, poultry, fish, nuts, legumes, and limiting the intake of low-fat dairy products, red meat, sweets, and sugar-containing beverages. The DASH diet provides higher potassium, calcium, magnesium, and protein while lowering total fat, saturated fat, and diet cholesterol (41). An excellent quantitative description of the design of this diet concept is presented by (42).

#### Pescatarian diet

This diet includes fish, dairy, and eggs but avoids all meats

#### Vegan diet

This sustainable diet concept does not contain any animal product. Instead, they are substituted by calcium-rich soy and extra portions of pulses. Protein sources for this diet design are similar to vegetarian. In addition, however, vegetable consumption is increased. A detailed description of this diet and the corresponding quantitative servings in proportion and key-related nutrients is presented by (43).

#### The healthy U.S style diet pattern

The healthy U.S diet style is recommended under the Diet Guidelines for Americans. This sustainable diet concept emphasizes consuming fruits, vegetables, whole grains, low- and fat-free dairy, healthy fat, lean meats, and poultry to reduce the risk to chronic diseases and meet daily nutrient needs. A detailed description of the diet recommendation and permissible quantity for different age groups in America is presented in 2020–2025 U.S.D.A Diet guidance (44).

#### Flexitarian diet concept

This sustainable diet concept can be regarded as a semi-vegetarian diet or perhaps a more plant-forward diet. Thus, the diet concept is less strict than a 100% vegetarian diet. The diet emphasizes incorporating plant-based foods and beverages, including eggs, meat, and dairy, into one's diet. However, it encourages a lower consumption quantity for meat and other dairy products (45).

#### Pro vegetarian food pattern

Pro vegetarian diet pattern has a preference for plant-derived foods but not the exclusion of animal foods. Its diet composition is similar to the vegetarian, howbeit the proportional intake of meat, vegetables and other food groups vary.

### Results of assessment

#### Weight of criteria

**Figure 8:**
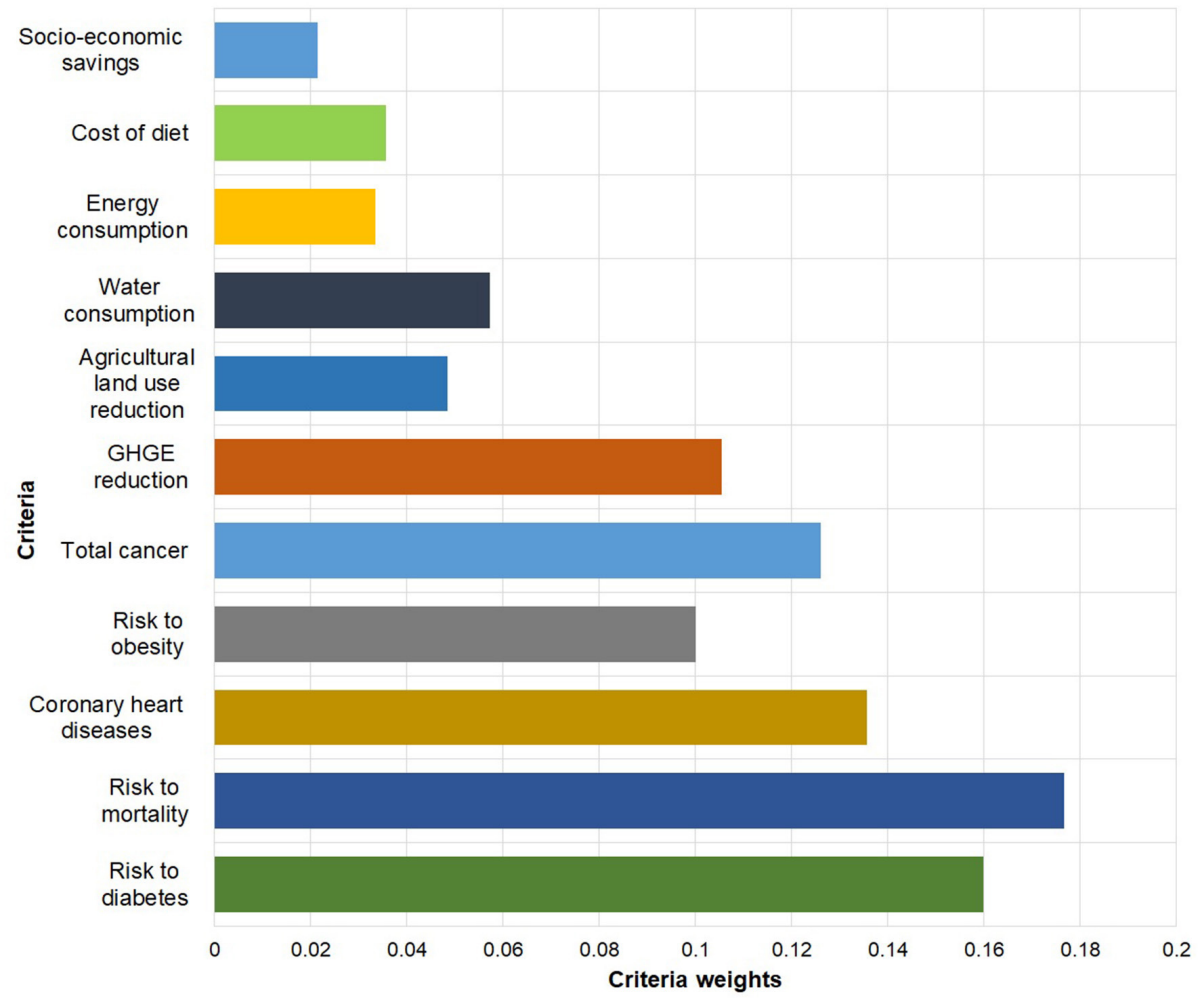
Weights of importance of criteria adopted to evaluate sustainable diet concepts.

Figure 8 represents the weights reflecting the relative importance of the evaluation criteria obtained from implementing the A.H.P. framework. One advantage of the technique is that it allows both qualitative and quantitative evaluation of criteria based on a preference scale.

Figure 8 shows that the risk of mortality (priority weight of = 0.18), coronary heart diseases (priority weight of 0.15), and diabetes was of a high priority compared to other environmental and socio-economic indicators. A possible explanation for these results may be attributed to the fact that health impacts have individual implications with selective socio-economic repercussions. Thus, participants in the survey could relate intimately/were familiar with these indicators compared to others. Another possible explanation is that the participants may have direct or personal experiences with the implication of these indicators, hence giving them a high priority. Nonetheless, we observe that the environmental indicators have a relatively lower weight; thus 0.11 and 0.0561 for GHGE and water consumption. The results may be explained by the fact that the implications of environmental indicators are collective while those of health are at an individual level.

To ascertain the validity of the results of the A.H.P framework, we conducted a consistency test. As a result, the final consistency index (CI) and consistency ratio were 0.14 and 0.091, respectively. Since the C.R. < 0.1, it indicates that the reliability of the responses from the participants could be maintained. The indicator matrix obtained from the survey and the eigenvalue of each criterion is reported in the Supplementary material.

#### Health and environmental impact evaluation results

**Figure 9:**
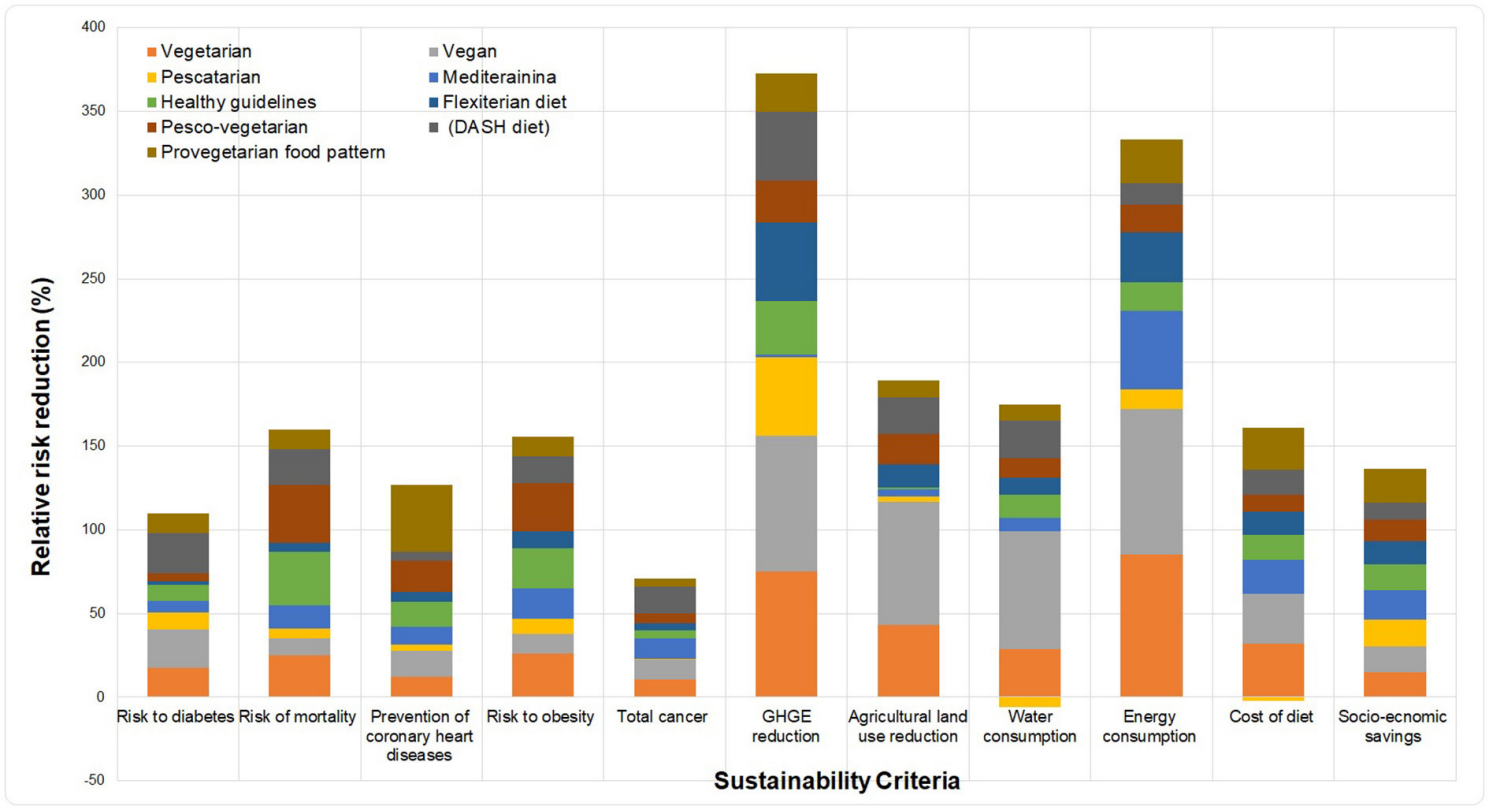
Relative reduction/increase in health and environmental impact of different dietary concepts.

Over the last half-century, multiple cohort studies have compared the health outcomes and environmental impact of different diet patterns such as Mediterranean, vegetarian, and vegan diets among individuals who consume them. A large portion of these studies has consistently observed a reduced risk of diabetes, heart diseases, hypertension, and total mortality compared to individuals on western or omnivorous diet patterns. In general, strict adherence to sustainable diet concepts offers significant health benefits. In addition, most studies have demonstrated that adopting healthier diets have some varying increased environmental sustainability. Figure 9 illustrates the relative reduction in health, socio-economic and environmental indicators by shifting current diet patterns to sustainable patterns.

It is observed from Figure 9 that a shift to sustainable diet concepts such as Vegetarian and Vegan diet would reduce the total GHGE, land use, water consumption, and energy use by (75.00, 81.40%), (74.98, 74.00%), (10.00, 70.00%), and (86.00, 87.00%), respectively. Furthermore, we observe a relative closeness in results due to the similar due to similar product composition that exists between the two diet concepts. Likewise, for health risk reduction, we observe that adherence to the Vegetarian and Vegan diet reduces the risk to diabetes, total mortality, heart diseases, obesity, and total cancer by (17.80, 19.30%), (25.00, 10.00%), (12.30, 15.10%), (26.00, 12.00%), and (10.40%, 11.70%), respectively. Shifting to other diet concepts such as the Mediterranean diet and the healthy US-style diet, we observe a relatively lower reduction compared to other diet concepts.

One of the surprising findings of the study was, adopting the U.S. diet style results in an average overall lower GHGE impact of 2% (reduction), water consumption of 14% (reduction), and an increase in energy consumption of 17% compared to other diet concepts. These results corroborate strongly with the studies of (46), who found a relative increase in GHGE from U.S. diet style.

It is important to mention that the results presented here are average values Life Cycle Assessment (L.C.A) studies on the selected diet concepts in the United States. The data collected for each diet pattern are isocaloric (equivalent in total calories).

#### Ranking from TOPSIS

**Figure 10:**
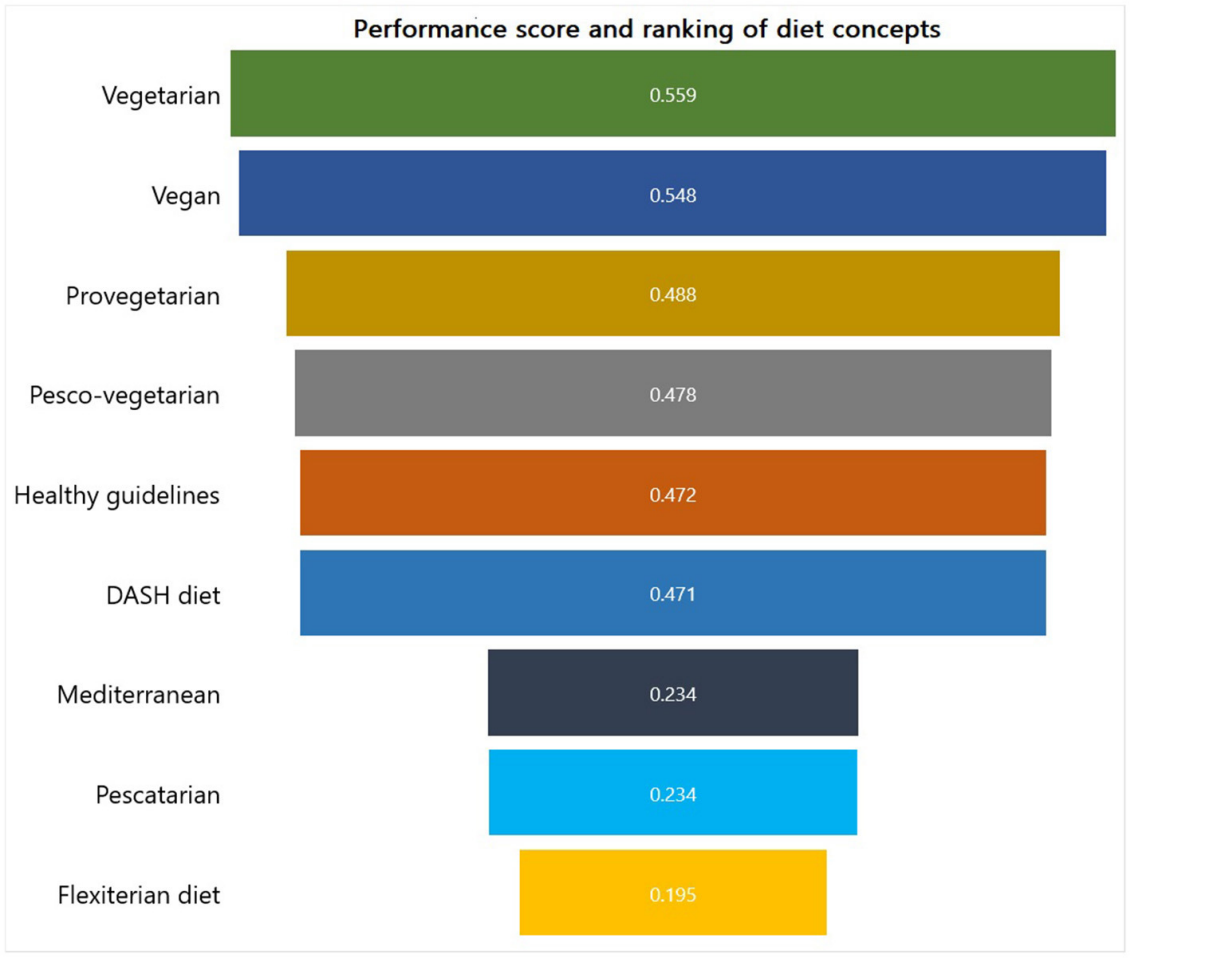
Ranking of dietary concepts using the TOPSIS framework.

Using the results obtained in sections Weight of criteria and Health and Environmental impact evaluation results, we ranked the diet concepts using the integrated AHP-TOPSIS decision model. From Figure 10, the Vegetarian, Vegan, and Provegetarian diets ranked first, second, and third, with a performance score of 0.553, 0.519, and 0.507, respectively. This is somewhat surprising as the vegan diet appears to have a better environmental impact reduction as compared to the vegetarian diet concept (see Section Health and Environmental impact evaluation results). On the contrary, the vegetarian diet has higher health impact reductions for some indicators as compared to the vegan diet concept. From a socioeconomic perspective, the vegetarian diet concept has a slightly higher reduction than the vegan diet. However, the model adopted for the evaluation takes into consideration the criteria weights presented in Section Weight of criteria. To wit, we observe from Figure 8 that higher weights were allocated to health indicators as compared to environmental and socio-economic indicators. Consequently, influencing the overall performance score and ranking of vegetarian and vegan diet concepts.

The results imply that adopting and national-wide implementation of different vegetarian diet concepts can substantially reduce diets' environmental and health impacts. Our results corroborate strongly with previous research of (8, 9, 15), who illustrated that the adoption of diets higher in plant-based than animal-based foods against the national Healthy US-style diet pattern would benefit the environment and the population's health. Furthermore, the results further reinforce previous research on the impact of diet on the environment and suggest that Vegetarian, Vegan, and Provegeterian diet pattern has the most sustainable impact on U.S. diet style. Despite these benefits, several bottlenecks and challenges exist that hinder the successful adoption of these concepts in America. The following Section explores different challenges, provides recommendations, and proposes a dynamic methodological framework to ensure a sustainable food system.

### Barriers in adoption and recommendations

So far, we have assessed which health, environmental and socio-economic factors are relevant to consumers, evaluated nine distinct sustainable diet concepts using sustainability metrics, and ranked these concepts to identify the optimal diet concept. Nonetheless, several challenges hinder the adoption and implementation of these sustainable diet concept. This Section identifies the bottlenecks in implementing different sustainable diet concepts and presents recommendations to rebuild a resilient and sustainable food system. Table 5 summarizes the challenges associated with adopting candidate sustainable diet concepts.

**Table 5:** Bottleneck to implementing sustainable diet concepts.

**S/N**	**Diet pattern**	**Bottleneck/critical challenges in adoption**	**Recommendation**
1	Vegetarian	•Risk of sub-optimal nutrients, including iron. •The iron source for most vegetarians is non-haem which has a lower bioavailability. •Consumer perception of vegetarian diet being expensive, not enjoyable, and inconvenient. •Reluctance in most consumers to try novel foods which they are unfamiliar with •Perceived nutritional need of meat (mostly as a source of protein), which is not provided. •Perceived difficulty in preparing plant-based food.	Early familiarization during childhood Informative, easy to read, and straightforward labeling on food products to alleviate food neophobia (Clean labeling).
2	Vegan	•The Diet concept is regarded as inconvenient since its products are challenging to prepare. Also, ingredients for preparing meals are most often unavailable in stores. •The perception that plant-based food does not taste better than animal-based food. •Meat consumption is highly ingrained in the culture of many hence the willingness to stop is difficult. •Consumer perception that plant-based milk substitutes have a similar environmental footprint as that of animals. •A general lack of awareness of the environmental burden of animal meat production and consumption.	Less processing of plant-based meat alternative with the intention of mimicking animal meat (reduced preservatives and sodium). Proper communication of diet benefits
3	Pescatarian	•Fear of the presence of heavy metals in food.	
4	Mediterranean diet	•Increase price of food items in the Mediterranean diet. •It promotes diet diversity, while diet recommendations suggest the consumption of healthier foods. •Socio-economic inequalities in domains such as diet variety, access to organic foods, and food purchasing behavior. •The vague idea of the overall diet framework. •Improper definition of the Mediterranean diet as different organizations and individual authors have presented a variety of diets labeled the Mediterranean.	Careful examination of the effectiveness of relying on a diet pyramid vs. promoting the health full aspects of individual foods that are included in the pyramid is needed Development of interventions to promote the diet
5	Healthy U.S. style guidelines	•Limited access to supermarkets and grocery stores. •Low-income	
6	DASH diet	•Poor availability, quality and cost of healthy and fresh food components within the diet. •Family influences and tension among family members' willingness to adopt a healthy diet. •Lack of familiarity with the DASH diet menu options. •The potential cost of preparing the recipes presented in the DASH diet was high. •Unfamiliarity with some of the menus found in the DASH diet appeared distracting.	Interventions to promote the DASH diet yet reflect the customer, economic and food available concern Optimized DASH diet with familiar recipes that conform the DASH diet pattern. Effective communication of DASH diet health information.

**Figure 11:**
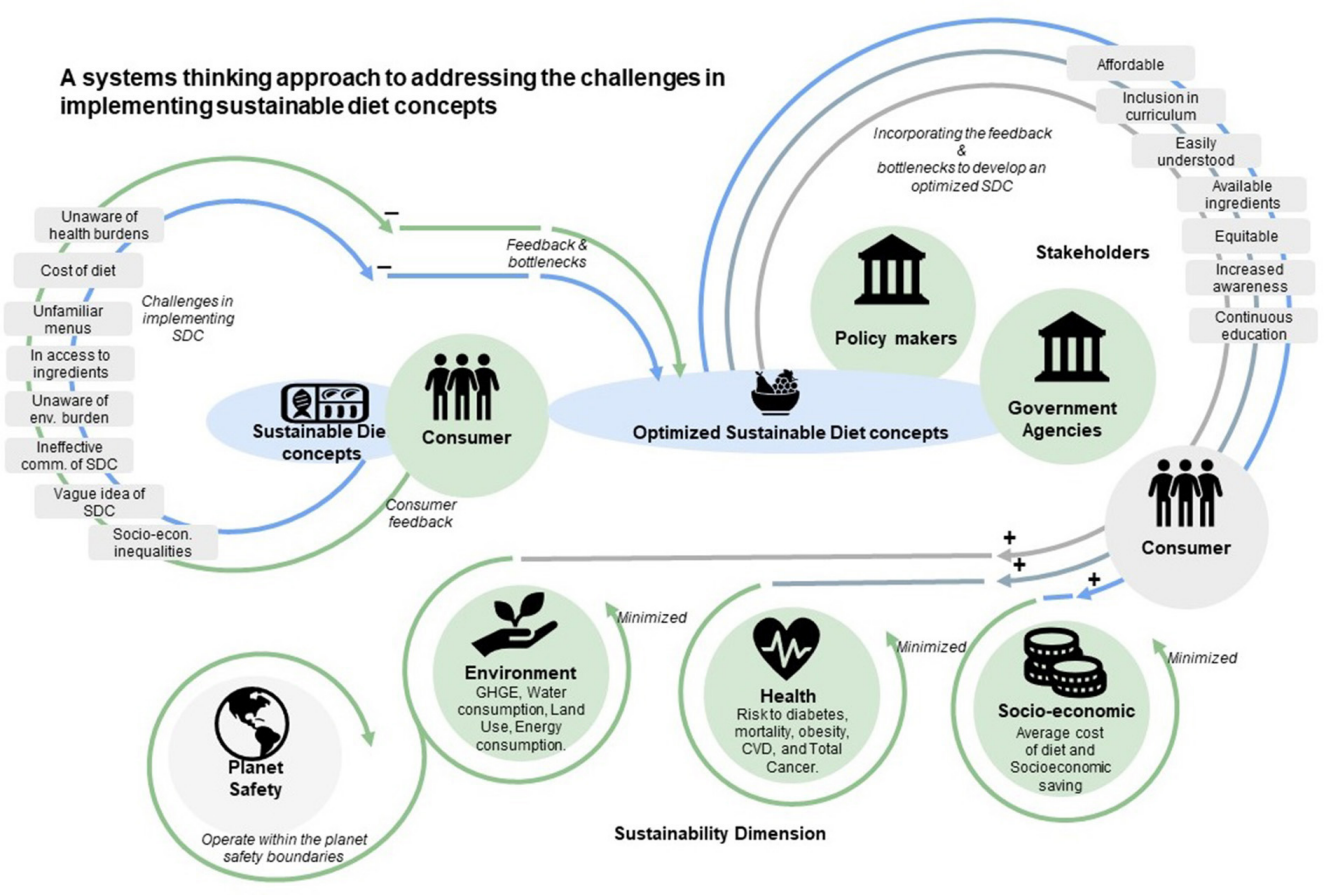
A system thinking approach to address the challenges of scaling up sustainable diet concepts to an optimized diet concept.

From Table 5, it is clear that widening the adoption of the sustainable diet concept presents a challenge, thus the need to understand the synergies in socio-economic, demographic, health, and environmental priorities. Sustainable diet concepts interact with consumer preference and wide array of social, economic and environmental systems, thus presenting a complex interaction driven by multiple factors. More importantly, a lack of information flow between the different actors and their respective systems exacerbate these shortcomings. Additional, knowledge on the trade-offs at varying Spatio-temporal scales is required; thus, we propose a conceptual system thinking approach for effective implementation of need. Figure 11 presents the conceptual framework that illustrates a holistic representation of sustainable diet concepts and their interconnections between actors, bottlenecks, components and different sub-systems. The elements in conceptual framework interact dynamically to give rise to predictable health, environmental and socio-economic impacts. The framework argues for a better and holistic integration of bottlenecks such as lack of knowledge and feedback across the interactions between the different components of the system and actors. Also, the framework argues for transparent sharing of information among actors to develop an optimized sustainable diet.

Application of system thinking and related tools can be found in different fields such energy, financial sectors and policy making. Increasingly, these different fields recognize the necessity of system thinking approaches to addressing today's interconnected challenges. Thus, the authors argue that the adoption of system thinking and related tools can help all actors of sustainable diet concepts to better plan for future interventions and wide adoption among consumers. Furthermore, policies can be enacted to introduce sustainable diet concepts to the population at an early childhood stage. It could be integrated into curriculums during early childhood education. Multi-sectoral efforts and campaigns from public organizations, local authorities, government, and non-governmental institutions to raise public awareness on the enormous benefits of sustainable diets will be paramount. Therefore, the proposed system thinking approach seeks to navigate stakeholders in implementation sustainable diet concepts toward a more comprehensive and broader picture by considering all interconnected factors to achieve a systemic change.

### Limitations of the study

The novel framework also suggests that optimized sustainable diet concepts that take into consideration multiple conflicting objectives as well their trade-offs have the potential to address the diet- health-environment trilemma. One major limitation of this study is that the authors observed a moderate variability in life cycle assessment results despite considering similar diet concepts. These may be attributed to the choice of parameters, the definition of system boundaries, the decision of function units, and the uncertainty evaluation adopted during the assessment. More disturbingly, most of these life cycle assessment studies do not account for the type of agroecology which may improve the environmental outcomes.

## Conclusion

The present study set out to evaluate the effectiveness of the implementation of sustainable healthy diet concepts in the United States. The study also examined the relationship between sustainable diet concepts and key factors that lead to improvement in human health, reductions in environmental damage and socio-economic benefits. Additionally, the AHP framework applied by the authors, provided an opportunity to curate expert opinions on which environmental-health-socio-economic indicators were of outermost relevance when considering resource allocation to optimize the adoption of sustainable diet concepts. The findings indicate that health indicators such as risk to mortality and cardiovascular disease are highly prioritized compared to other socio-economic, and environmental indicators. Through the application of mathematical modeling (AHP-TOPSIS) and a set of environmental, health and socio-economic indicators, vegetarian, vegan and provegetarian diet concepts ranked first, second and third, respectively. The findings provide additional evidence that sustainable diet concepts which constitute more plant-based than animal-based foods are more beneficial to the environmental and population's health as compared to the national Healthy US-style diet concept which has an average overall lower GHGE impact of 2% (reduction), water consumption of 14% (reduction), and an increase in energy consumption of 17% compared to other diet concepts. However, the implementation and wider adoption of sustainable diet concepts is hindered by intrinsic socio-economic, cultural and behavioral barriers. These include a lack of understanding, limited access to food ingredients, and unfamiliarity with sustainable diet menus. Hence, the study proposed a novel conceptual system thinking framework to sustainable diet concepts, which takes into consideration these bottlenecks prior to implementation sustainable diets on larger scale. The proposed can potentially optimize sustainable diet acceptance by consumers and offset different health, environmental and socio-economic impacts. The novel framework shows the complex interactions and dynamics between diet concepts, social cultural challenges, food environment, key stakeholders and multiple subsystems. Taken together, it provides a holistic representation of optimizing sustainable diet initiatives and adoption among consumers. It would be interesting to assess the effectiveness of the conceptual system thinking approach through a practical application of system dynamic models, then translate the results through an intervention case study.

## Data Availability Statement

The original contributions presented in the study are included in the article/Supplementary material, further inquiries can be directed to the corresponding author/s.

## Author contributions

PA: conceptualization, investigation and expert survey, methodology, data curation, writing (initial draft), and data visualization. EK: conceptualization, investigation and survey, resources, writing (review and editing), and supervision. JB: conceptualization, methodology, and writing (review and editing). All authors contributed to the article and approved the submitted version.

## Funding

This project was funded by the Graduate Professional Student Congress Tiffany Marcantonio Research Grant at the University of Arkansas and Department of Biological and Agricutural Engineering, University of Arkansas.

## Conflict of interest

The authors declare that the research was conducted in the absence of any commercial or financial relationships that could be construed as a potential conflict of interest.

## Publisher's note

All claims expressed in this article are solely those of the authors and do not necessarily represent those of their affiliated organizations, or those of the publisher, the editors and the reviewers. Any product that may be evaluated in this article, or claim that may be made by its manufacturer, is not guaranteed or endorsed by the publisher.

## References

[1] Garnett T. Where are the best opportunities for reducing greenhouse gas emissions in the food system (including the food chain)?. Food Policy. (2011) 36:S23–32. 10.1016/j.foodpol.2010.10.010

[2] Phan K Kothari P Lee NJ Virk S Kim JS Cho SK. Impact of obesity on outcomes in adults undergoing elective posterior cervical fusion. Spine. (2017) 42:261–6. 10.1097/BRS.0000000000001711 28207668

[3] Phillips JA. Dietary guidelines for Americans, 2020–2025. Workplace Health Saf. (2021) 69:395. 10.1177/21650799211026980 34279148

[4] CDC A.W. Centers for Disease Control and Prevention. Chicago (2020).

[5] Chai BC van der Voort JR Grofelnik K Eliasdottir HG Klöss I Perez-Cueto FJ. Which diet has the least environmental impact on our planet? A systematic review of vegan, vegetarian and omnivorous diets. Sustainability. (2019) 11:4110. 10.3390/su11154110

[6] Domingo NG Balasubramanian S Thakrar SK Clark MA Adams PJ Marshall JD. Air quality–related health damages of food. Proc Nat Acad Sci USA. (2021) 118:e2013637118. 10.1073/pnas.2013637118 33972419 PMC8158015

[7] Willett W Rockström J Loken B Springmann M Lang T Vermeulen S. Food in the anthropocene: the EAT–Lancet Commission on healthy diets from sustainable food systems. Lancet. (2019) 393:447–92. 10.1016/S0140-6736(18)31788-4 30660336

[8] Burlingame B Dernini S. Biodiversity and Sustainable Diets United Against Hunger 3–5 November 2010. Rome: FAO Headquarters (2012).

[9] Agyemang P Kwofie EM. Response-to-failure analysis of global food system initiatives: a resilience perspective. Front Sustain Food Syst. (2021) 208. 10.3389/fsufs.2021.676997

[10] Reinhardt SL Boehm R Blackstone NT El-Abbadi NH McNally Brandow JS Taylor SF. Systematic review of dietary patterns and sustainability in the United States. Adv Nutr. (2020) 11:1016–31. 10.1093/advances/nmaa026 32167128 PMC7360461

[11] Mekonnen MM Fulton J. The effect of diet changes and food loss reduction in reducing the water footprint of an average American. Water Int. (2018) 43:860–70. 10.1080/02508060.2018.1515571

[12] Orlich MJ Singh PN Sabaté J Jaceldo-Siegl K Fan J Knutsen S. Vegetarian dietary patterns and mortality in Adventist Health Study 2. JAMA Intern Med. (2013) 173:1230–8. 10.1001/jamainternmed.2013.6473 23836264 PMC4191896

[13] Springmann M Clark MA Rayner M Scarborough P Webb P. The global and regional costs of healthy and sustainable dietary patterns: a modelling study. Lancet Planetary Health. (2021) 5:e797–807. 10.1016/S2542-5196(21)00251-5 34715058 PMC8581186

[14] Fresán U Martínez-González MA Sabaté J Bes-Rastrollo M. Global sustainability (health, environment and monetary costs) of three dietary patterns: Results from a Spanish cohort (the SUN project). BMJ Open. (2019) 9:e021541. 10.1136/bmjopen-2018-021541 30796113 PMC6398639

[15] Blackstone NT El-Abbadi NH McCabe MS Griffin TS Nelson ME. Linking sustainability to the healthy eating patterns of the Dietary Guidelines for Americans: a modelling study. Lancet Planet Health. (2018) 2:e344–52. 10.1016/S2542-5196(18)30167-0 30082049

[16] Aleksandrowicz L Green R Joy EJ Smith P Haines A. The impacts of dietary change on greenhouse gas emissions, land use, water use, and health: a systematic review. PLoS ONE. (2016) 11:e0165797. 10.1371/journal.pone.0165797 27812156 PMC5094759

[17] Schwingshackl L Missbach B König J Hoffmann G. Adherence to a Mediterranean diet and risk of diabetes: a systematic review and meta-analysis. Public Health Nutr. (2015) 18:1292–9. 10.1017/S1368980014001542 25145972 PMC10273006

[18] Zhong X Guo L Zhang L Li Y He R Cheng G. Inflammatory potential of diet and risk of cardiovascular disease or mortality: A meta-analysis. Sci Rep. (2017) 7:1–6. 10.1038/s41598-017-06455-x 28744020 PMC5527022

[19] Reedy J Krebs-Smith SM Miller PE Liese AD Kahle LL Park Y. Higher diet quality is associated with decreased risk of all-cause, cardiovascular disease, and cancer mortality among older adults. J Nutr. (2014) 144:881–9. 10.3945/jn.113.189407 24572039 PMC4018951

[20] Behrens G Gredner T Stock C Leitzmann MF Brenner H Mons U. Cancers due to excess weight, LOW physical activity, and unhealthy diet: Estimation of the attributable cancer burden in Germany. Deutsches Ärzteblatt Int. (2018) 115:578. 10.3238/arztebl.2018.0578 30236216 PMC6206246

[21] Clark M Hill J Tilman D. The diet, health, and environment trilemma. Annu Rev Environ Resour. (2018) 43:109–34. 10.1146/annurev-environ-102017-025957

[22] Röös E Carlsson G Ferawati F Hefni M Stephan A Tidåker P. Less meat, more legumes: prospects and challenges in the transition toward sustainable diets in Sweden. Renew Agric Food Syst. (2020) 35:192–205. 10.1017/S1742170518000443

[23] Macdiarmid JI Kyle J Horgan GW Loe J Fyfe C Johnstone A. Sustainable diets for the future: can we contribute to reducing greenhouse gas emissions by eating a healthy diet? Am J Clin Nutr. (2012) 96:632–9. 10.3945/ajcn.112.038729 22854399

[24] Lewis M McNaughton SA Rychetnik L Lee AJ. Cost and affordability of healthy, equitable and sustainable diets in low socioeconomic groups in Australia. Nutrients. (2021) 13:2900. 10.3390/nu13082900 34445059 PMC8402089

[25] Ritchie H Roser M. Environmental Impacts of Food Production. Cambridge, MA: Our World in Data (2020).

[26] FAOSTAT. Food and Agricultural data. Available from: https://www.fao.org/faostat/en/#home (accessed January 21, 2022).

[27] Clark MA Springmann M Hill J Tilman D. Multiple health and environmental impacts of foods. Proc Nat Acad Sci USA. (2019) 116:23357–62. 10.1073/pnas.1906908116 31659030 PMC6859310

[28] Johnston JL Fanzo JC Cogill B. Understanding sustainable diets: a descriptive analysis of the determinants and processes that influence diets and their impact on health, food security, and environmental sustainability. Adv Nutr. (2014) 5:418–29. 10.3945/an.113.005553 25022991 PMC4085190

[29] Wang JJ Jing YY Zhang CF Zhao JH. Review on multi-criteria decision analysis aid in sustainable energy decision-making. Renew Sustain Energy Rev. (2009) 13:2263–78. 10.1016/j.rser.2009.06.021

[30] Neba FA Agyemang P Ndam YD Emmanuel E Ndip EG Seidu R. Leveraging integrated model-based approaches to unlock bioenergy potentials in enhancing green energy and environment. Green Energy Environ. (2020) 89:109–134. 10.5772/intechopen.91978

[31] Saaty TL. Modeling unstructured decision problems: A theory of analytical hierarchy. In: Proceedings of the First International Conference on Mathematical Modeling. Vol. 1 (1997). p. 59–77.

[32] National Research Council. Diet and Health: Implications for Reducing Chronic Disease Risk. Basel: National Academies Press (1989). 25032333

[33] Arnich N Sirot V Rivière G Jean J Noël L Guérin T. Dietary exposure to trace elements and health risk assessment in the 2nd French Total Diet Study. Food Chem Toxicol. (2012) 50:2432–49. 10.1016/j.fct.2012.04.016 22521625

[34] Tasevska N Jiao L Cross AJ Kipnis V Subar AF Hollenbeck A. Sugars in diet and risk of cancer in the NIH-AARP Diet and Health Study. Int J Cancer. (2012) 130:159–69. 10.1002/ijc.25990 21328345 PMC3494407

[35] Afshin A Sur PJ Fay KA Cornaby L Ferrara G Salama JS. Health effects of dietary risks in 195 countries, 1990–2017: a systematic analysis for the Global Burden of Disease Study 2017. Lancet. (2019) 393:1958–72.28. 10.1073/pnas.190690811 30954305 PMC6899507

[36] Obesity Collaborators. Health effects of overweight and obesity in 195 countries over 25 years. N Engl J Med. (2017) 377:13–27. 10.1016/S0140-6736(19)30041-8 28604169 PMC5477817

[37] Lozano R Naghavi M Foreman K Lim S Shibuya K Aboyans V. Global and regional mortality from 235 causes of death for 20 age groups in 1990 and 2010: a systematic analysis for the Global Burden of Disease Study 2010. Lancet. (2012) 380:2095–128. 10.1016/S0140-6736(12)61728-0 23245604 PMC10790329

[38] Westhoek H Lesschen JP Rood T Wagner S De Marco A Murphy-Bokern D. Food choices, health and environment: effects of cutting Europe's meat and dairy intake. Global Environ Change. (2014) 26:196–205. 10.1016/j.gloenvcha.2014.02.004

[39] Vieux F Darmon N Touazi D Soler LG. Greenhouse gas emissions of self-selected individual diets in France: changing the diet structure or consuming less? Ecol Econ. (2012) 75:91–101. 10.1016/j.ecolecon.2012.01.003

[40] Stallones RA. Patterns of Health Risk: Seven Countries,. A, Multivariate Analysis of Death and Coronary Heart, Disease., Ancel Keys with 15 others. Harvard University, Press, Cambridge, Mass., 1980. xvi, 382 p, illus. $25. A Commonwealth Fund Book. Science. (1980) 208:1138–9. 10.1126/science.208.4448.1138 17783068

[41] Steinberg D Bennett GG Svetkey L. The DASH diet, 20 years later. JAMA. (2017) 317:1529–30. 10.1001/jama.2017.1628 28278326 PMC5509411

[42] Kwan MWM Wong MCS Wang HHX Liu KQL Lee CLS Yan BPY. Compliance with the dietary approaches to stop hypertension (DASH) diet: a systematic review. PLoS ONE. (2013) 8:e78412. 10.1371/journal.pone.0078412 24205227 PMC3813594

[43] Craig WJ Mangels AR. Position of the American Dietetic Association: vegetarian diets. J Am Diet Assoc. (2009) 109:1266. 10.1016/j.jada.2009.05.027 19562864

[44] You A. Dietary Guidelines for Americans. New England: US Department of Health and Human Services and US Department of Agriculture (2015). 7 p.

[45] Grasso S Jaworska S. Part meat and part plant: are hybrid meat products fad or future? Foods. (2020) 9:1888. 10.3390/foods9121888 33348859 PMC7767298

[46] Birney CI Franklin KF Davidson FT Webber ME. An assessment of individual foodprints attributed to diets and food waste in the United States. Environ Res Lett. (2017) 12:105008. 10.1088/1748-9326/aa8494

